# Strontium Doping Promotes Low-Temperature Growth of Single-Crystalline Ni-Rich Cathodes with Enhanced Electrochemical Performance

**DOI:** 10.3390/ma18061320

**Published:** 2025-03-17

**Authors:** Jiaqi Wang, Yunchang Wang, Mengran Zheng, Feipeng Cai

**Affiliations:** 1 Energy Research Institute, Qilu University of Technology (Shandong Academy of Sciences), Keyuan Road 19, Jinan 250014, China; wjq980223@163.com (J.W.); 17569153983@163.com (Y.W.); zhengmengran888@163.com (M.Z.); 2 Jinan Key Laboratory of Advanced Energy Storage and Hydrogen Utilization, Jinan 250014, China

**Keywords:** nickel-rich cathode, li-ion batteries, single-crystal growth, SrO dopant

## Abstract

Nickel-rich cathode materials have emerged as ideal candidates for electric vehicles due to their high energy density; however, polycrystalline materials are prone to microcrack formation and unavoidable side reactions with electrolytes during cycling, leading to structural instability and capacity degradation. Herein, an Sr-doped single-crystalline nickel-rich LiNi_0.88_Co_0.05_Mn_0.07_O_2_/Sr cathode material is synthesized, with Sr doping levels controlled at x = 0.3%, 0.5%, 1 mol%. The nickel-rich LiNi_0.88_Co_0.05_Mn_0.07_O_2_/Sr cathode features particle sizes of approximately 2 μm, at a relatively low temperature. It inhibits the microcrack formation, prevents electrolyte penetration into the particle interior, and reduce side reactions, thereby enhancing structural stability. This enables the cathode to deliver a high initial discharge capacity of 205.3 mAh g^−1^at 0.1 C and 170.8 mAh g^−1^ at 10 C, within the voltage range of 2.7 V–4.3 V, and an outstanding capacity retention of 96.61% at 1 C after 100 cycles. These improvements can be attributed to the Sr-doping, which reduces the single-crystal growth temperature, effectively mitigating Li^+^/Ni^2+^ cation mixing. Moreover, the incorporation of Sr expands the interlayer spacing, thereby facilitating Li^+^ diffusion. The doping strategy employed in this work provides a new insight for low-temperature single-crystal materials synthesis, significantly improving the electrochemical performance of nickel-rich cathode materials.

## 1. Introduction

Lithium ion batteries (LIBs), celebrated for their exceptional electrochemical properties—especially their high energy density and extended cycle life—have become indispensable in the development of next-generation energy solutions and electric vehicles [1,2]. As a critical component of LIBs, cathode materials contribute to 30–40% of the total cost, underscoring their significance in research [3]. LiNi_x_Co_y_Mn_1-x-y_O_2_ (NCM) cathode materials, particularly those with high nickel content (x > 0.8), have attracted substantial research interest, owing to their outstanding capacity [4]. However, high nickel content cathode materials face several critical challenges that must be addressed, chief among them being the excessive Ni content, which readily results in the formation of a high concentration of Ni^4+^ ions during charging. The interaction between Ni^4+^ ions and the electrolyte facilitates the formation of rock-salt phases. This phenomenon may trigger the collapse of the layered structure and promote microcrack formation [5,6]. Compared to Li^+^, Ni^2+^ exhibits an almost identical ionic radius. During battery operation, Ni^2+^ readily migrates to Li^+^ sites, occupying Li^+^ lattice positions, disrupting the layered structure, and ultimately leading to lithium–nickel mixing [7,8].

Elemental doping and single-crystallization, widely recognized as effective strategies for overcoming the aforementioned challenges, have been extensively investigated in academia [9]. Doping effectively alleviates irreversible phase transitions and suppresses microcrack formation. Proper bulk doping substantially enhances the electrochemical performance of materials [10]. By harnessing surface reactivity, Nb doping promotes the formation of an electrochemically active protective layer on the grain surface, effectively mitigating electrolyte corrosion and suppressing adverse phase transitions. Furthermore, Nb doping mitigates Li^+^/Ni^2+^ cation mixing, thereby enhancing Li^+^ diffusion kinetics [11]. Due to the stronger bond strength of P–O interactions, P doping enhances phase stability, mitigates phase transitions, and suppresses microcrack formation. B doping significantly improved structural stability, achieving a high capacity retention of 91.35% [12,13]. Wang et al. [14] optimized the primary particle morphology of NCA90 via Hf doping, inducing the formation of densely packed, radially aligned short rod-like structures, which enhanced the toughness of the secondary particles and minimized internal porosity. Hf doping enhanced the structural stability and mitigated side reactions through the formation of stable Hf−O bonds. Hf^4+^ effectively stabilizes the crystal structure and modulates particle morphology, thereby enhancing cycling stability. The synergistic benefits of Hf doping substantially enhanced the cycling stability of NCA90 Hf0.5, yielding a remarkable capacity retention of 95.3% after 100 cycles at 1 C. Single-crystal cathode materials possess larger primary crystal grains, with highly ordered internal atomic arrangements and fewer defects at grain boundaries and intergranular regions. This structure allows single-crystal materials to more effectively withstand volume changes in usage, thereby minimizing the occurrence of microcracks and particle fractures, thus improving the overall structural stability [15,16]. Additionally, single-crystal materials reduce side reactions at grain boundaries, slow down impedance growth, and extend the cycle life and safety performance of batteries [17]. Compared to polycrystalline cathode materials, single-crystals exhibit significant advantages in structural stability and performance. However, preparing single-crystal cathode materials presents substantial hurdles. Single-crystal growth typically requires high-temperature environments, but prolonged high-temperature calcination can lead to severe Li/Ni mixing and an inferior electrochemical performance [18,19]. Consequently, synthesizing single-crystal cathode materials at low temperatures has become a research focus. Many researchers have employed the molten salt-assisted method to grow single-crystal ternary cathode materials at low temperatures [20]; however, some researchers have found that the incorporation of Ce can also reduce the growth temperature of single-crystal materials [21]. Compared to the molten salt method, the use of element doping alone to promote single-crystal growth can reduce subsequent steps such as washing. Lee successfully synthesized single-crystal cathode materials through Ce doping at lower temperatures. The results indicated that capacity retention could be improved by 10% at lower calcination temperatures, with larger primary grains when Ce is doped. Additionally, the cycling performance is enhanced due to a reduction in phase transitions and fewer microcracks [22].

With the increase in nickel content, obtaining ideal single-crystal materials at lower temperatures becomes increasingly challenging in comparison to Ni80. In this study, Sr-doped single-crystal LiNi_0.88_Co_0.05_Mn_0.07_O_2_/Sr cathode material was designed and synthesized to address defects arising from excessively high single-crystal growth temperatures. Sr doping enables the growth of single-crystal grains to 2 μm at relatively low temperatures and with a more uniform particle size. Larger single-crystal particles can efficiently mitigate microcrack formation, prevent electrolyte infiltration into the particle interior, and suppress side reactions. Moreover, Sr doping strengthens the crystal structure and minimizes structural changes and lattice distortions during charge–discharge cycles, thereby enhancing cycling stability. The Sr-doped LiNi_0.88_Co_0.05_Mn_0.07_O_2_/Sr composite demonstrates an exceptional capacity retention of 96.61% after prolonged cycling, significantly outperforming the 88.48% observed in the undoped counterpart. Notably, the incorporation of Sr enhances structural stability, likely through the modulation of lattice parameters. The Sr-doping strategy presents a promising approach for optimizing single-crystalline nickel-rich ternary cathodes, offering enhanced electrochemical performance and structural integrity.

## 2. Experimental

### Preparation of Materials

**Figure 1:**
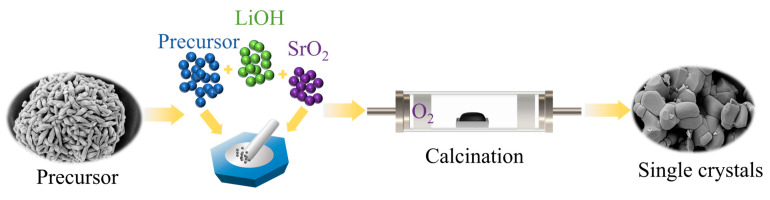
Flowchart of the single-crystal preparation process.

As shown in Figure 1. The commercial precursor Ni_0.88_Co_0.05_Mn_0.07_(OH)_2_ from Zibo Kesimo Youbang New Materials Co., Ltd. (Zibo, China) was thoroughly mixed with an excess lithium ratio of 1.03 LiOH·H_2_O (98% purity, purchased from Kesimo) and SrO (99.99% purity, purchased from Macklin, Germering, Germany), at molar ratios of 0.3%, 0.5%, and 1%. The blended powder material was subsequently transferred into a tube furnace for sintering, where the furnace atmosphere was sustained under a constant flow of pure oxygen throughout the entire sintering process. The blended powder material was then placed in a tube furnace for sintering, with the furnace maintained under a continuous flow of pure oxygen throughout the sintering process. Initially, the material was calcined at 550 °C for 5 h to completely remove moisture and melt the LiOH. It was then calcined at 790 °C, 820 °C, and 850 °C for 10 h, respectively, to ensure a complete reaction, resulting in the formation of LiNi_0.88_Co_0.05_Mn_0.07_O_2_/Sr single-crystal cathode material. Conversely, the polycrystalline material (PC-NCM) was sintered under controlled conditions, specifically with a Li/TM ratio of 1.01 and at a temperature of 760 °C. Materials without SrO doping were labeled as PC-NCM, NCM-790, NCM-820, and NCM-850, respectively. SrO-doped materials were sintered at the optimal calcination temperature of 790 °C for 10 h, resulting in LiNi_0.88_Co_0.05_Mn_0.07_O_2_/Sr doped with Sr at concentrations of 0.3%, 0.5%, and 1%, named NCM-0.3Sr, NCM-0.5Sr, and NCM-1Sr, respectively. The details of material characterization and electrochemical testing are provided in the Supplementary Materials.

## 3. Results and Discussion

### Material Characterization

As shown in Table 1, the LiNi_0.88_Co_0.05_Mn_0.07_O_2_/Sr material was first analyzed using ICP (Inductively Coupled Plasma) testing. The data in the table indicate that the elemental composition of Ni, Co, and Mn in the synthesized precursor material closely aligns with the expected molar ratio of 0.88:0.05:0.07 [23].

**Figure 2:**
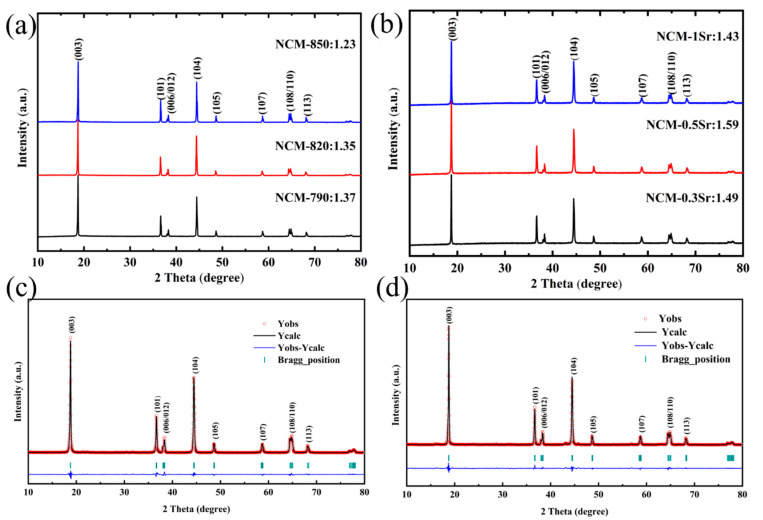
(**a**,**b**) Show the XRD patterns of NCM-790, NCM-820, NCM-850, NCM-0.3Sr, NCM-0.5Sr, and NCM-1Sr; (**c**,**d**) display the refined patterns of NCM-790 and NCM-0.5Sr.

As shown in Figure 2a,b, the X-ray diffraction (XRD) peaks of all Sr-doped samples closely resemble those of the undoped material, indicating that Sr doping does not alter the intrinsic structure of the material, which remains the rhombohedral α-NaFeO_2_ layered structure with the space group R-3m [24]. The diffraction peaks of samples with different Sr ratios are sharp, and no impurity peaks are observed, demonstrating that all samples possess an excellent crystalline quality. The distinct separation of the I(006)/I(102) and I(018)/I(110) peaks highlights the well-ordered layered structure present in all three Sr-doped samples [25]. All doped materials exhibit I(003)/I(104) ratios exceeding 1.2, indicating that the extent of cation mixing in Sr-doped samples is minimal [26]. Calculations reveal that the corresponding values for the three samples first increase and then decrease, with values of 1.49, 1.59, and 1.43. Excessive Sr doping may affect the material’s lattice properties, which in turn affect the arrangement and interaction of ions within the material, causing an uneven distribution of cations in the crystal structure. This uneven distribution may intensify cation mixing, which could potentially account for the initial decline followed by a subsequent rise in the I(003)/I(104) ratio [27]. Nevertheless, the ratio of cation mixing in the three materials decreased compared to the value of 1.37 observed in NCM-790. Excessive cation mixing can lead to decreased electrochemical performance, reduced structural stability, and impaired safety; however, with the occupation of Sr in the lattice, it can reduce the lithium–nickel mixing, thereby strengthening the crystal structure and minimizing structural changes during cycling, which enhances the cyclic stability of the material. Figure 2c,d presents the outcomes of the additional Rietveld refinement applied to the XRD data, as shown in Table 2. All c/a ratios exceed 4.9, indicating a well-ordered layered structure of the material [28]. With the incorporation of Sr, the lattice parameters of a, c, and the unit cell volume all increase, as the ionic radius of Sr^2+^ is 1.18 Å, which is larger than that of the three transition metal ions in the material. Therefore, when Sr^2+^ replaces Ni^3+^, a charge imbalance occurs in the lattice. To compensate for the charge imbalance, Ni^3+^ may partially reduce to Ni^2+^, thereby balancing the charge distribution. This reduction of Ni^3+^ to Ni^2+^ may relieve lattice stress to some extent, improving the structural stability of the material and thus extending the cycle life of the battery. Under high voltage or heavy load conditions, many transition metal oxide materials are prone to irreversible phase transitions, leading to capacity degradation or performance decline in the battery. The introduction of Sr^2+^ can suppress these phase transitions, as its larger radius and stable valence state help to stabilize the lattice structure, thus reducing the likelihood of phase changes. Finally, the NCM-0.5Sr sample exhibits a widened TM layer and a contracted Li layer compared to NCM-790. An increase in the width of the transition metal layer typically indicates enhanced structural stability, while the compactness of the lithium layer may suggest a more ordered migration pathway for lithium ions. This ordering facilitates improved conductivity and diffusion of lithium ions, thereby enhancing the cycling performance of the battery.

**Figure 3:**
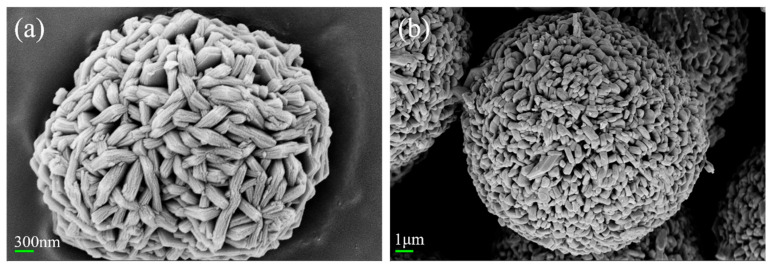
(**a**) SEM image of the precursor; (**b**) SEM image of PC-NCM.

As illustrated in Figure 3a, the SEM images of the commercial precursor Ni_0.88_Co_0.05_Mn_0.07_(OH)_2_ show that the precursor is made up of spherical secondary particles that are created when many flaky primary particles aggregate, which are still nanoscale in size. As shown in Figure 3b, the polycrystalline material particles remain as agglomerated spherical particles. This is due to insufficient temperature, preventing complete sintering and significant growth of the primary particles.

**Figure 4:**
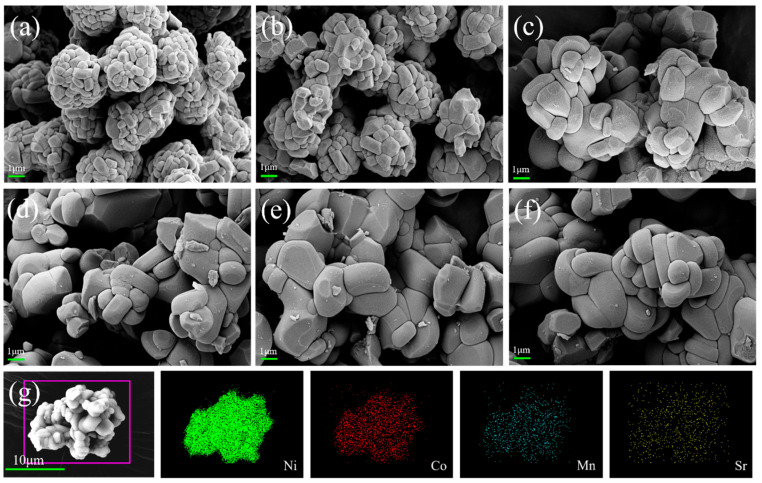
(**a**–**c**) SEM images of NCM-790, NCM-820, and NCM-850; (**d**–**f**) SEM images of NCM-0.3Sr, NCM-0.5Sr, and NCM-1Sr; (**g**) EDS mapping SEM image of NCM-0.5Sr.

In Figure 4a–f, the SEM images are labeled as NCM-790, NCM-820, NCM-850, NCM-0.3Sr, NCM-0.5Sr, and NCM-1Sr, respectively. From Figure 4a–c, we can clearly see the grain morphology at different temperatures. The three sets of single-crystal samples exhibit significantly larger grain sizes compared to the polycrystalline materials. As the temperature increases, further growth of grains is evident, especially in the sample at 850 °C, where the grain size grows to 1–2 μm. However, uneven particle distribution is also observed. Compared to polycrystalline samples, the primary particles in the NCM-790 and NCM-820 samples show significant growth, but the outline of precursor spherical particles is still visible, and their dispersion is relatively moderate. In Figure 4d–f, the calcination temperature is consistently 790 °C; however, the three Sr-added materials exhibit significant growth compared to the non-added samples, reaching a grain size of about 2 μm, even at relatively lower temperatures. The agglomeration of Sr-doped samples is greatly reduced, indicating that the introduction of Sr can affect the crystal growth rate, allowing single-crystal materials to form at lower temperatures. More stable Sr-doped material structures can result from larger primary particle sizes because they can lessen the side interactions between the material and the electrolyte. This stability helps to reduce the structural damage caused by high temperatures or cycling during battery use, thereby improving the battery’s safety performance [29]. The dispersibility of the NCM-0.3Sr and NCM-0.5Sr samples was also improved, with agglomerates generally consisting of only a few particles, indicating that the material’s agglomeration was not severe. However, the agglomeration degree of NCM-1Sr significantly increased again, and the particle size was not uniform. The increased dispersion of particles enhances the contact area between the active components within the electrode and the electrolyte, thereby improving material utilization and subsequently augmenting the energy density of the battery. As illustrated in Figure 4g, the surface element distribution was analyzed using EDS mapping. The elements of Ni, Co, Mn, and Sr exhibited uniform distribution throughout the material [30]. Based on the results of the current research, it is possible to deduce that Sr doping can effectively increase the grain surface structure stability, hence improving material safety.

**Figure 5:**
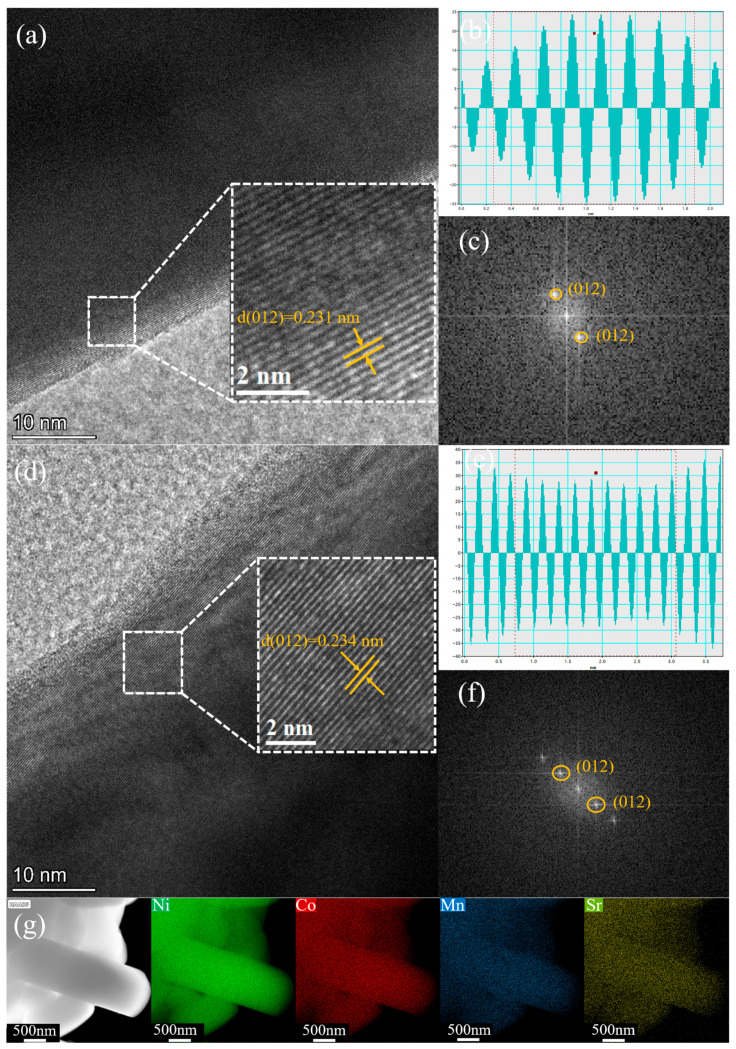
(**a**–**c**) Represent the HRTEM images, lattice stripe spacing images, and SAED patterns of NCM-790, respectively; (**d**–**f**) represent the HRTEM images, lattice stripe spacing images, and SAED patterns of NCM-0.5Sr, respectively; (**g**) shows the EDS mapping of the elemental distribution in NCM-0.5Sr.

The structure of the material was analyzed using transmission electron microscopy (TEM). Figure 5a–f shows that lattice fringes can be clearly observed for NCM-790 and NCM-0.5Sr. Fourier transform and lattice spacing measurements were conducted on NCM-790 and NCM-0.5Sr, yielding lattice spacings of 0.231 nm and 0.234 nm, which correspond to the (012) plane alignment of the LiNiO_2_ layered structure. The Fourier transform findings match to the characteristic diffraction sites of the (012) plane [31]. NCM-0.5Sr exhibits an ordered layered structure throughout both the surface and internal regions, characterized by a larger lattice spacing compared to NCM-790, which aligns with the results of the XRD refinement. The incorporation of Sr elongates the c-axis of the crystal structure [32]. According to Figure 5g, the TEM image of NCM-0.5Sr shows an ideal single-crystal morphology, and EDS results indicate that Sr is uniformly doped into the grains, consistent with the above results. Based on the findings, it is possible to deduce that the addition of Sr can increase the material’s electrochemical performance while also promoting crystal structure stability.

**Figure 6:**
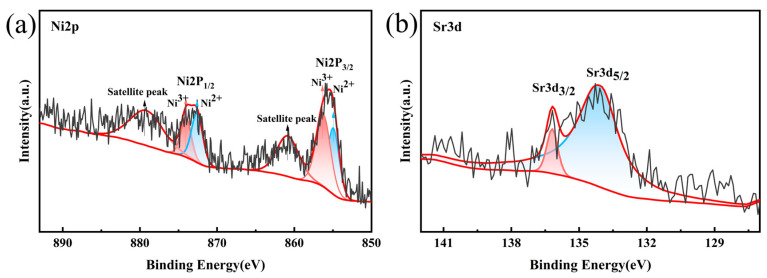
(**a**) Shows the NCM-0.5Sr Ni2p XPS spectrum and (**b**) shows the Sr3d XPS spectrum.

The surface chemical valence states of the single-crystal material were investigated by utilizing XPS. As shown in Figure 6a, the Ni2p orbital was decomposed into two peaks, Ni2P_3/2_ (855.48 eV) and 2P_1/2_ (873.38 eV). These peaks fit well with the Ni2p orbital [33]. The Ni2P_3/2_ displays two characteristic peaks at 856.24 eV and 855.06 eV, which correspond to the two distinct valence states of nickel, which are as follows: Ni^2+^ and Ni^3+^ [34]. The integration of peak areas shows that the peak area of Ni^3+^ is larger than that of Ni^2+^, indicating the existence of additional Ni^3+^ on the sample surface. The reduction in Ni^2+^ content can decrease Li^+^/Ni^2+^ mixing, which corroborates the XRD refinement results [35]. The data shown in Figure 6b reveal that the two characteristic peaks of Sr3d_3/2_ and Sr3d_5/2_ at 136.18 eV and 134.18 eV verify the successful incorporation of Sr into the lattice. Sr doping reduces the formation of lithium compounds on the surface of the grains, leading to a more stable structure.

**Figure 7:**
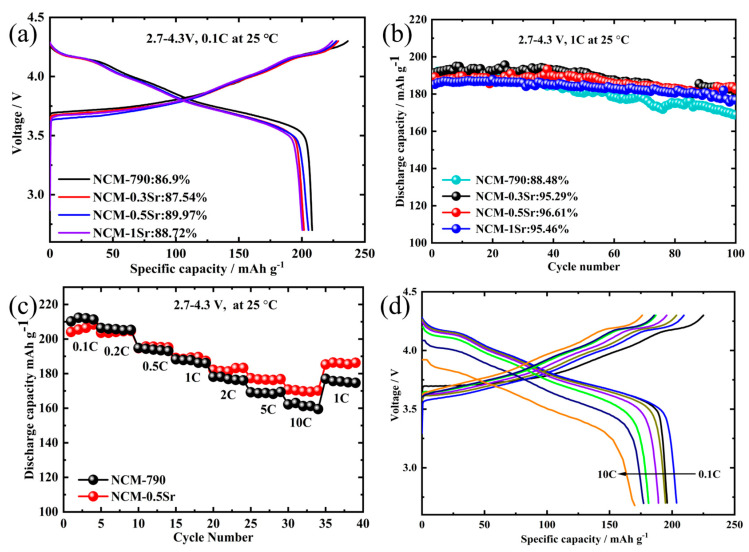
(**a**) Shows the initial charge–discharge curves at 0.1 C; (**b**) illustrates the cycling performance at 1 C; (**c**) presents the rate performance diagram of NCM-790 and NCM-0.5Sr at different rates; (**d**) displays the initial discharge capacity distribution of NCM-0.5Sr at different rates.

As shown in Figure 7a, at 0.1 C, the initial discharge capacities of NCM-790, NCM-0.3Sr, NCM-0.5Sr, and NCM-1Sr are 210.1 mAh g^−1^, 201.7 mAh g^−1^, 205.3 mAh g^−1^, and 200.4 mAh g^−1^, respectively, with first-cycle Coulombic efficiencies of 86.9%, 87.54%, 89.97%, and 88.72%. The discharge curves for all materials are nearly identical, indicating that Sr doping does not significantly alter the intrinsic structure of the materials. The incorporation of Sr facilitates the efficient synthesis of micron-sized single-crystal cathode materials at a lower temperature of 790 °C. However, the discharge-specific capacity of Sr-doped materials is slightly lower compared to undoped samples and further decreases as the amount of Sr doping increases. Identifying an appropriate doping level is essential for achieving an optimal electrochemical performance. Sr-doped materials can effectively suppress voltage decay and structural transitions that may occur during cycling. Volume changes during lithium ion extraction and insertion can potentially lead to structural fracturing and capacity loss; furthermore, Sr exhibits remarkable structural stability, facilitating the reversible insertion and extraction of lithium ions, thereby maintaining the structural integrity and cycling stability of the cathode material [36]. This stability ensures that the battery maintains its performance over an extended period, thereby extending its lifespan.

Figure 7b shows the cycling data of the Sr-doped sample over 100 cycles at 1 C. The initial discharge capacities of NCM-790, NCM-0.3Sr, NCM-0.5Sr, and NCM-1Sr are 191.3 mAh g^−1^, 191.4 mAh g^−1^, 189.1 mAh g^−1^, and 185.2 mAh g^−1^, respectively. After 100 cycles, the capacity retention rates of the materials were 88.48%, 95.29%, 96.61%, and 95.46%, respectively. The test results show that the cycling performance of the Sr-doped materials is significantly better than the 88.48% observed for the NCM-790 sample. Figure 7c shows the rate performance of the material, with five charge–discharge cycles conducted at different rates. NCM-790 exhibited a capacity of 210.2 mAh g^−1^ at 0.1 C and 162.2 mAh g^−1^ at 10 C, with a retention of 77.16%. NCM-0.5Sr demonstrated a capacity of 204.1 mAh g^−1^ at 0.1 C and 170.8 mAh g^−1^ at 10 C, with a retention rate of 83.68%. Compared to NCM-790, NCM-0.5Sr exhibits a pronounced reduction in the discharge-specific capacity at 0.1 C and a slight decrease at 1 C; however, at elevated charge rates, the capacity of Sr-doped single-crystal materials significantly surpasses that of undoped counterparts. Additionally, Sr-doped materials exhibit better capacity retention across different rates. The discharge performance of Sr-doped materials is more stable at varying rates because Sr doping stabilizes the lattice structure, thus delaying the phase transition process and enhancing material stability [37].

**Figure 8:**
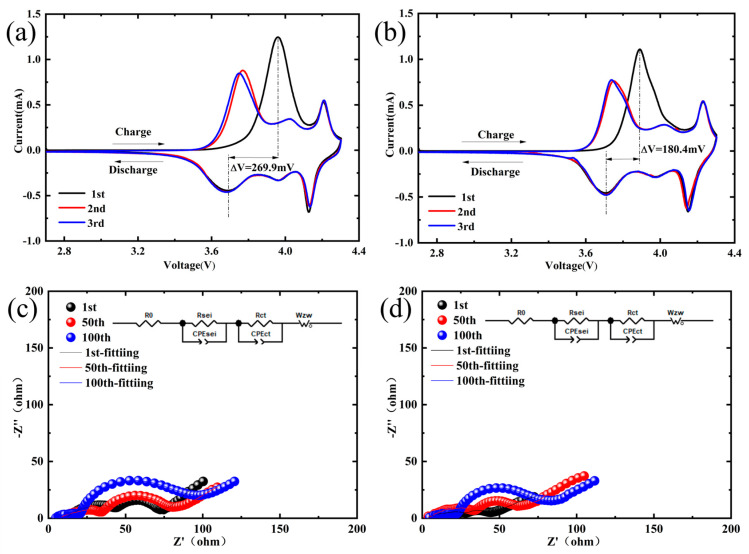
(**a**,**b**) Show the CV curves of NCM-790 and NCM-0.5Sr materials; (**c**,**d**) are the electrochemical impedance plots of NCM-790 and NCM-0.5Sr materials after 1, 50, and 100 cycles.

CV testing was conducted on the material. Figure 8a,b presents the test results, depicting the CV curves for NCM-790 and NCM-0.5Sr, respectively. The similar shapes of the CV curves for the two materials indicate that the Sr doping did not alter the intrinsic reactions within the material. All samples exhibited three pairs of redox peaks in their curves, indicating that during charge and discharge processes, multiple redox reactions and phase transitions occurred at different voltages [38]. During oxidation, both single-crystal materials showed one large oxidation peak and two smaller oxidation peaks, reflecting the transition from H1 to M, M to H2, and H2 to H3 in the material [39,40]. The potential difference for NCM-790 is 269.9 mV, while for NCM-0.5Sr it is approximately 180.4 mV. The reduction in the potential difference suggests that the introduction of Sr may also affect the electron distribution and charge state within the cathode material, influencing the ease of electron transfer, thereby enhancing the diffusion rate of lithium ions. This change is reflected as a smaller potential difference on the CV curve. As lithium ion diffusion becomes easier, polarization phenomena during charge and discharge processes are alleviated [41,42]. The reduction in the potential difference also indicates that Sr doping can enhance the cyclic reversibility of the material. This is consistent with the previously described high capacity retention results for Sr-doped samples.

Figure 8c,d, EIS tests were conducted on NCM-790 and NCM-0.5Sr materials during cycling to obtain the surface impedance variations of different materials. Initially, the NCM-790 and NCM-0.5Sr materials underwent activation for three cycles at 0.1 C, by 100 cycles at 1 C. Subsequently, the coin half-cells were subjected to electrochemical impedance spectroscopy (EIS) testing after three, fifty, and one-hundred cycles [43]. Table 3 summarizes the test results for the ohmic resistance (R_o_), surface film resistance (R_SEI_), and charge transfer resistance (R_ct_) of the tested materials. Throughout the cycling, the R_o_ of all samples was less than 3 Ω, which can be considered negligible. As cycling progressed, the R_SEI_ of each sample gradually decreased, indicating the gradual formation and stabilization of the SEI film within the material. Meanwhile, R_ct_ gradually increased, which could lead to a reduction in reaction rate and capacity fade [44,45]. The R_ct_ of NCM-790 is 39.403 Ω in the first cycle, which increases to 46.101 Ω in the 50th cycle and reaches 79.463 Ω by the 100th cycle. The R_ct_ of NCM-790 was 39.403 Ω in the first cycle, which increased to 46.101 Ω in the 50th cycle and reached 79.463 Ω in the 100th cycle. The charge transfer resistance of the Sr-doped sample significantly decreased in each cycle, particularly reaching 57.914 Ω in the 100th cycle, a reduction of approximately 27.12% compared to NCM-790. This indicates that Sr doping can reduce charge transfer resistance, thereby accelerating electron transport within the battery, reducing energy loss, and consequently enhancing cycle efficiency. Furthermore, Sr doping further increased the primary particle size at the same temperature.

**Figure 9:**
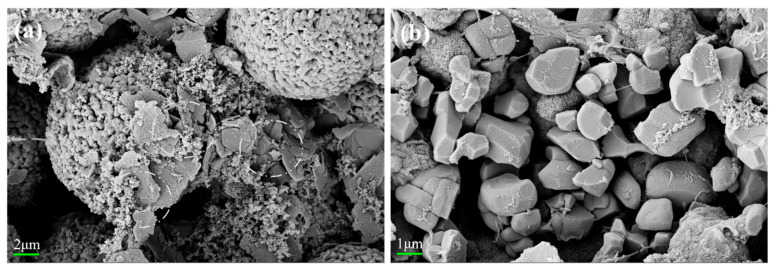
(**a**,**b**) Show the SEM images of PC-NCM and NCM88-0.5Sr after 100 cycles of charge and discharge.

After 100 weeks of cycling at 1 C current, the electrode materials were analyzed using SEM. In Figure 9a, the secondary particles of PC-NCM exhibit significant particle fracture and microcracks. This may cause the electrolyte to infiltrate the particles along the fissures and stimulate additional side reactions, resulting in TM dissolution and crystal structure degradation [20,46]. In contrast, the single-crystal NCM-0.5Sr material shown in Figure 9b exhibits excellent integrity in its primary particle structure, with larger primary particles effectively preventing microcracks and ensuring that the electrolyte cannot penetrate into the particles, thereby reducing side reactions. Sr^2+^ doping increases the particle size while ensuring that the material exists in the form of dispersed micron-sized primary particles. This structural feature makes the internal structure of the particles more uniform and stable. Such uniformity reduces the stress concentration and cracking caused by volume changes during charge and discharge. This structural feature makes the internal structure of the particles more uniform and stable, which can help reduce the stress concentration and cracking caused by volume changes during cycling, thereby improving the structural stability of the material. Therefore, the single-crystal NCM-0.5Sr material exhibits a superior performance in terms of electrochemical properties.

## 4. Conclusions

This study introduces an Sr-doping strategy to synthesize single-crystal LiNi_0.88_Co_0.05_Mn_0.07_O_2_/Sr cathode materials. Sr doping not only lowers the synthesis temperature, but also facilitates the formation of single-crystal structures approximately 2 μm in size. The presence of larger single-crystal particles effectively mitigates microcrack formation, prevents electrolyte penetration into the particles, and suppresses undesired side reactions. After 100 cycles at 1 C within a voltage range of 2.7–4.3 V, NCM-0.5Sr retains 96.61% of its initial capacity, significantly outperforming the undoped NCM-790 sample (88.48%). The NCM-0.5Sr sample delivers an impressive discharge capacity of 170.8 mAh g^−1^ at 10 C, surpassing the 162.2 mAh g^−1^ recorded for the undoped NCM-790 sample. Sr incorporation enhances the structural stability of the material by modifying lattice parameters, thereby mitigating structural degradation during cycling. Furthermore, Sr doping reduces charge transfer resistance, enhances electron transport within the battery, minimizes energy loss, and improves both cycling stability and rate capability. The Sr-doping strategy markedly enhances electrochemical performance and offers a novel avenue for low-temperature single-crystal growth.

## Figures and Tables

**Table 1:** Compositional analysis of LiNi_0.88_Co_0.05_Mn_0.07_O_2_/Sr.

Sr-Doped Composite Materials	Ni (mol%)	Co (mol%)	Mn (mol%)	Sr (mol%)
LiNi_0.88_Co_0.05_Mn_0.07_O_2_/Sr	88.13	4.83	7.31	0.496

**Table 2:** The lattice parameters of NCM-790 and NCM-0.5Sr after refinement.

Sample	a	c	c/a	V(Å3)	R(I003/I104)	Ni in Li Sites (%)	TM Slab	Li Slab
NCM-790	2.8696	14.1740	4.9404	101.03	1.37	1.5	2.1263	2.5990
NCM-0.5Sr	2.8718	14.1947	4.9429	101.38	1.59	1.23	2.1331	2.5984

**Table 3:** The fitted Ro, RSEI, and Rct values for NCM-790 and NCM-0.5Sr.

Sample	NCM-790			NCM-0.5Sr		
	R_o_(Ω)	R_SEI_(Ω)	R_ct_(Ω)	R_o_(Ω)	R_SEI_(Ω)	R_ct_(Ω)
Cycling						
1st	1.833	34.182	39.403	2.304	30.707	23.429
50th	2.657	32.016	46.101	2.443	28.448	32.506
100th	2.891	18.807	79.463	2.755	22.599	57.914

## Data Availability

The original contributions presented in this study are included in the article and Supplementary Materials. Further inquiries can be directed to the corresponding author.

## Supplementary Materials

The following supporting information can be downloaded at: https://www.mdpi.com/article/10.3390/ma18061320/s1, Figure S1: Displays the initial discharge capacity distribution of NCM-790 at different rates; Figure S2: Atomic Percentages of Elements by EDS.
