# Supplementary material for: Strontium Doping Promotes Low-Temperature Growth of Single-Crystalline Ni-Rich Cathodes with Enhanced Electrochemical Performance
Source: Materials (Basel). 2025 Mar 17;18(6):1320. doi: 10.3390/ma18061320 (PMC11943760; doi:10.3390/ma18061320)
Supplement: Supplementary file 1 [file materials-18-01320-s001.zip › materials-3526456-supplementary.pdf]

Strontium Doping Promotes Low-Temperature Growth of Single-Crystalline Ni-Rich Cathodes with Enhanced Electrochemical Performance

Jiaqi Wang [1,2], Yunchang Wang [1,2], Mengran Zheng [1,2] and Feipeng Cai [1,2,*]

1 Energy Research Institute, Qilu University of Technology (Shandong Academy of Sciences), Keyuan Road 19, Jinan 250014, China; wjq980223@163.com (J.W.); 17569153983@163.com (Y.W.); zhengmengran888@163.com (M.Z.)

2 Jinan Key Laboratory of Advanced Energy Storage and Hydrogen Utilization, Jinan 250014, China

* Correspondence: caifp@sderi.cn

Experimental

Material characterization

The chemical composition of the cathode material was analyzed using inductively coupled plasma spectroscopy (ICP). The crystal structure of the $LiNi_{0.88}Co_{0.05}Mn_{0.07}O_2$/Sr material was tested using X-ray diffraction (XRD) with Cu Kα radiation over a scanning range of 10°–80°. The morphology, structural properties, and elemental distribution of the material were comprehensively examined using Scanning Electron Microscopy (SEM) and High-Resolution Transmission Electron Microscopy (HRTEM). Additionally, the chemical valence states and surface chemical environments of the samples were probed using X-ray Photoelectron Spectroscopy (XPS, Thermo VG ESCALAB250).

Electrochemical measurements

The cathode materials, including conductive carbon black (Super P) and polyvinylidene fluoride (PVDF), were uniformly mixed in a mass ratio of 8:1:1, and N-

methyl-2-pyrrolidone was added to prepare the slurry. The prepared slurry was uniformly coated onto an aluminum current collector using a doctor blade, followed by drying in a vacuum oven at 90 °C for 12 hours, and then sliced into electrodes with a slicer. CR2032 button cells were assembled within a glove box under a high-purity argon atmosphere. All electrochemical tests were conducted in a controlled environment at 25 °C, spanning a voltage range of 2.7 V to 4.3 V, using the LAND testing system to assess the cycling and rate performance of the battery. The cycling test commenced with three cycles at a rate of 0.1 C, followed by a 100-cycle evaluation under conditions of 0.5 C charging and 1 C discharging. The rate test comprised charge–discharge cycling assessments for five cycles at rates of 0.1 C, 0.2 C, and 0.5 C, followed by five cycles of charge–discharge testing at 0.5 C charging and discharging rates of 1 C, 2 C, 5 C, and 10 C. Cyclic voltammetry (CV) and electrochemical impedance spectroscopy (EIS) tests were performed using the Zahner IM6 electrochemical workstation, manufactured by Zahner. The CV test was conducted at a scan rate of 0.1 $mV \cdot s^{-1}$, while the EIS test frequency ranged from 10 MHz to 1 MHz, with a disturbance amplitude of 5 mV.

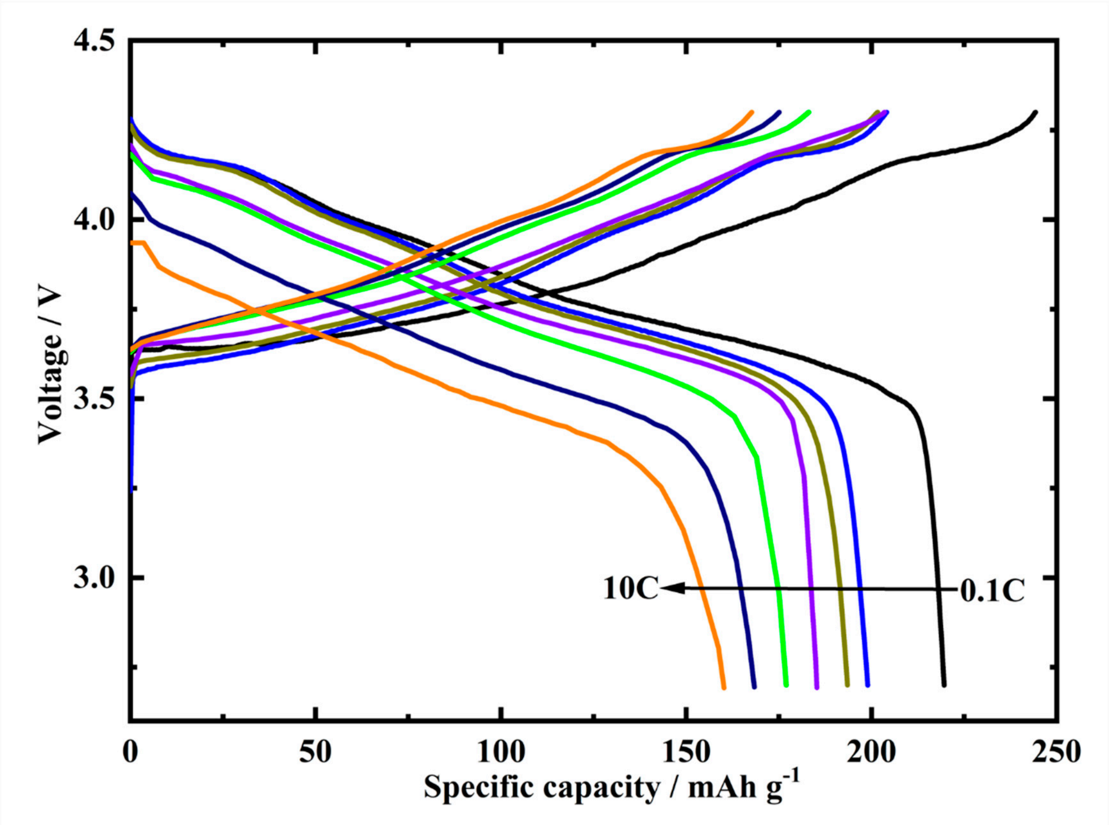

Figure S1 displays the initial discharge capacity distribution of NCM-790 at different rates.

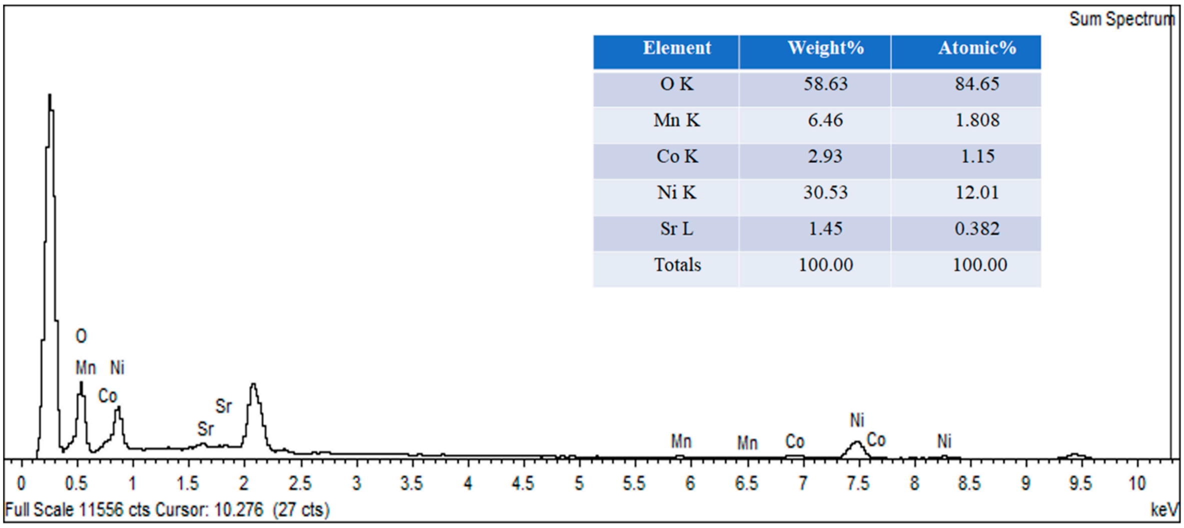

Element	Weight%	Atomic%
O K	58.63	84.65
Mn K	6.46	1.808
Co K	2.93	1.15
Ni K	30.53	12.01
Sr L	1.45	0.382
Totals	100.00	100.00

Figure S2 Atomic Percentages of Elements by EDS
